# In Vitro Models of Tissue and Organ Regeneration

**DOI:** 10.3390/ijms241914592

**Published:** 2023-09-26

**Authors:** Patrick C. Baer, Ralf Schubert

**Affiliations:** Division of Allergology, Pneumology and Cystic Fibrosis, Department for Children and Adolescents, University Hospital, Goethe-University, 60596 Frankfurt/M., Germany

The recovery of cells after tissue and organ injury is a complex process. To understand the underlying molecular biological mechanisms, more detailed insights into the cellular processes of repair and regeneration are urgently needed. Based on this knowledge, this Special Issue focuses on current in vitro systems exploring repair and regeneration mechanisms. Experimental research approaches to investigate the mechanisms involved and laboratory methods to establish and optimise models for tissue and organ repair and regeneration, as well as theoretical modelling and computational models, but also review papers are included here. Eleven articles are published in the Special Issue, which deals with various tissue and organ regeneration questions or the modelling or summary of the research models used in this process.

Shyam et al. comprehensively summarise various methods involved in developing 3D cell culture systems, emphasising the differences between 2D and 3D systems and methods involved in recapitulating the organ-specific 3D microenvironment [1]. They also discuss the latest developments in 3D tissue model fabrication techniques, microfluidics-based organ-on-a-chip, and imaging as a characterisation technique for 3D tissue models. Lieto et al. summarise current research to accurately evaluate ocular toxicity and drug effectiveness [2]. The recent achievements in tissue engineering of in vitro 2D, 2.5D, 3D, organoid and organ-on-chip ocular models and in vivo and ex vivo ocular models were discussed in terms of their advantages and limitations. Another review by Young et al. looks in detail at different strategies for fatty liver treatment in non-alcoholic fatty liver disease [3]. They discuss various defatting strategies, including in vitro use of pharmacologic agents, machine perfusion of extracted livers, and genomic approaches targeting specific proteins. Another work by Hentabli et al. deals with modelling a neural network for bioactivity prediction [4]. This paper describes a novel technique based on a deep learning convolutional neural network for predicting chemical compounds’ bioactivity. The authors explain the importance of this work by stating that determining and modelling the possible behaviour and effects of molecules requires the study of the basic structural features and physicochemical properties that determine their behaviour in chemical, physical, biological and environmental processes.

Two original works use in vitro models of mesenchymal stromal/stem cells (MSCs) to investigate regenerative purposes. Barbon and coworkers use an in vitro conditioning regimen of MSCs towards the endothelial lineage to stimulate coagulation factor VIII production [5]. The background of this work was the development of a cell therapy for the treatment of Haemophilia A and, therefore, for future pre-clinical investigation using preconditioned MSCs. Leppik and coworkers demonstrate a new perspective on bone tissue engineering [6]. The work shows that MSCs survive cryopreservation on scaffolds and, after thawing, could be released as ready-to-use products for permanent implantation during surgery. 

In addition, two other in vitro studies use epithelial cell systems to investigate their differentiation or their involvement in inflammatory processes. Primary alveolar epithelial cells’ main limitation is the difficulty of maintaining the type II phenotype in culture. Marhuenda and coworkers show that culturing primary alveolar epithelial cells on lung extracellular matrix-derived hydrogels facilitated the prolonged culturing of these cells and enhanced the preservation of the type II phenotype [7]. Baer and coworkers characterised the mRNA expression of renal proximal tubular epithelial cells and the cargo in extracellular vesicles in an inflammatory microenvironment [8]. This study demonstrates the altered miRNA expression of epithelial cells and their released vesicles during induced inflammation, with only three miRNAs overlapping between cells and vesicles. The background to this study is that understanding the precise molecular and cellular mechanisms that lead to inflammation is the most important way to identify targets for the prevention or treatment of inflammation.

Steyn-Ross and coworkers describe the ex vivo quantification of tissue oxygen consumption by measuring oxygen partial pressure as a function of probe depth using thin slices of cortical brain tissue [9]. The authors confirm that a previously published diffusion-consumption model provides an excellent description of the oxygen-tension distribution in a thin slice of active tissue.

Finally, this Special Issue contains two publications addressing issues using in vivo models. Azam and coworkers investigated the prevention of neuroinflammation in vitro and in vivo using an herbal extract and purified dioscin [10]. The in vitro study demonstrates protection against lipopolysaccharide-activated inflammatory responses in microglial cells. The following in vivo study shows that dioscin upregulates brain-derived neurotrophic factor and cAMP-response element binding protein phosphorylation in the cerebral cortex and hippocampus regions of the mouse brain. The authors conclude that dioscin protects against neurotoxicity. Esteves-Monteiro and coworkers evaluated changes in ileum and colon histomorphometry and Angiotensin II reactivity in a rat model of diabetes mellitus. They showed the structural remodelling of the gut wall with a decreased contractile response to Angiotensin II [11]. They summarise that these findings may help to explain diabetic dysmotility.
